# Transfer Effect of Speech-sound Learning on Auditory-motor Processing of Perceived Vocal Pitch Errors

**DOI:** 10.1038/srep13134

**Published:** 2015-08-17

**Authors:** Zhaocong Chen, Francis C. K. Wong, Jeffery A. Jones, Weifeng Li, Peng Liu, Xi Chen, Hanjun Liu

**Affiliations:** 1 Department of Rehabilitation Medicine, The First Affiliated Hospital, Sun Yat-sen University, Guangzhou, 510080, China; 2 Department of Rehabilitation Medicine, The Third Affiliated Hospital, Sun Yat-sen University, Guangzhou, 510630, China; 3 Division of Linguistics and Multilingual Studies, School of Humanities and Social Sciences, Nanyang Technological University, 14 Nanyang Drive, HSS-03-49, 637332, Singapore; 4 Psychology Department and Laurier Centre for Cognitive Neuroscience, Wilfrid Laurier University, Waterloo, Ontario, N2L 3C5, Canada

## Abstract

Speech perception and production are intimately linked. There is evidence that speech motor learning results in changes to auditory processing of speech. Whether speech motor control benefits from perceptual learning in speech, however, remains unclear. This event-related potential study investigated whether speech-sound learning can modulate the processing of feedback errors during vocal pitch regulation. Mandarin speakers were trained to perceive five Thai lexical tones while learning to associate pictures with spoken words over 5 days. Before and after training, participants produced sustained vowel sounds while they heard their vocal pitch feedback unexpectedly perturbed. As compared to the pre-training session, the magnitude of vocal compensation significantly decreased for the control group, but remained consistent for the trained group at the post-training session. However, the trained group had smaller and faster N1 responses to pitch perturbations and exhibited enhanced P2 responses that correlated significantly with their learning performance. These findings indicate that the cortical processing of vocal pitch regulation can be shaped by learning new speech-sound associations, suggesting that perceptual learning in speech can produce transfer effects to facilitating the neural mechanisms underlying the online monitoring of auditory feedback regarding vocal production.

A fundamental question for neuroscientists is how sensory information is integrated into the control of ongoing motor behaviour. Speech production is a particularly complex motor behaviour, and researchers have investigated not only the role of the auditory system for speech motor control but also the involvement of the motor system in speech perception^1^. It has been well documented that the availability of auditory feedback is important for the development and maintenance of accurate speech production^2^,^3^,^4^,^5^. For example, children’s speech development is compromised when hearing impairments eliminate or reduce the quality of their auditory feedback during speech^6^. This reliance on auditory feedback continues through adulthood, as laboratory studies have shown that unexpected alterations in auditory feedback during speech elicit compensatory vocal adjustments^2^,^3^,^7^,^8^, and brain imaging studies have shown that hearing altered auditory feedback (AAF) is associated with enhanced activity in auditory regions such as the superior temporal gyrus (STG) and superior temporal sulcus (STS)^4^,^9^,^10^.

On the other hand, the motor theory of speech perception posits that the speech motor system is crucial for not only the production of speech, but for the perception of speech as well^11^,^12^. Evidence for this notion comes from imaging studies that have shown that speech motor regions of the brain are active while individuals listen to speech sounds^13^,^14^. The discovery of mirror neurons that are active when individuals perform an action and listen to the sounds that are produced by that action^15^, suggests that the motor system may be involved in the perception of auditory stimuli produced by voluntary movements in general. Together, this growing body of literature indicates that perception and production of speech are intimately linked, and shares some neural processes and representations^16^.

Considering the speech perception-production link, the finding that improved speech fluency is matched by an increased ability of discriminating different speech sounds during language learning^17^, suggests that speech motor learning may be able to drive changes to the auditory processing of speech. Recently, several behavioural studies have assessed whether speech motor adaptation to auditory and somatosensory feedback perturbations has an impact on speech perception. For example, Nasir and Ostry^18^ asked participants to undergo a somatosensory-based motor training in which a robot device was used to displace their jaw during the production of the words *bad*, *had*, *mad*, *sad*. The participants who underwent the training perceived a synthesized speech stimulus along a *head-had* continuum more frequently as *head*, a perceptual shift away from the trained vowel stimulus, whereas participants who did not undergo the training did not show this perceptual shift. Similarly, Lametti *et al.*^19^ reported changes in the perceptual boundary between *head* and *had* as a result of speech motor adaptation to altered auditory feedback during the production of *head*. These perceptual after effects that result from sensorimotor adaptation are not limited to the perception of vowels. For example, Shiller *et al.*^20^ reported that, following the period of speech motor adaptation training under feedback manipulation of shifting the fricative centroid frequency of sibilant /s/ words lower towards /ʃ/, participants exhibited a shift in their perceptual identification boundary of the /s-ʃ/ contrast toward the direction of the /ʃ/ category. Thus, there is ample evidence that speech motor learning can lead to changes to the perceptual processing of speech.

Much less is known, however, about whether auditory perceptual learning can drive changes to the speech motor control. In the domain of limb motor control, there are several studies that have assessed the effect of perceptual learning on motor function^21^,^22^,^23^,^24^. For example, when subjects were required to indicate the direction (left or right) that their arm was passively moved by a robotic device along a set of fan-shaped trajectories (i.e. somatosensory discrimination training), the rate and extent of learning in a sensorimotor adaptation task increased compared to when participants were not asked to discriminate the movement of their arm^22^. In addition, changes in functional connectivity between the primary somatosensory cortex and the frontal motor areas were observed as a result of the somatosensory discrimination training^23^. These studies suggest that perceptual learning can benefit movement and motor learning, at least for limb movements.

In the context of speech processing, research on the second-language learning has shown changes in speech production as a consequence of perceptual training^25^,^26^. Recently, two behavioural studies combining a brief period of speech perceptual training with tests of speech motor adaptation to AAF have shown that changes in speech perception can affect speech production in the context of speech motor learning^19^,^27^. For example, 5- to 7-year old children who underwent an auditory training to perceive the /ε/-**/**æ/ contrast adapted significantly more to a 25% increase in the first formant (F1) of the vowel /ε/ compared to a group of children trained to perceive the /b/-/d/ contrast^27^. In adults, training-induced changes in the perceptual distinction between the words *bead* and *bad* cause immediate changes in the amount of speech motor learning as indexed by increased compensations for perceived F1 errors while producing the word *bead*^19^. There are then, a number of studies that indicate that perceptual learning of speech contrasts modifies speech motor control. However, the studies conducted thus far have focused on the perception and production of vowel formants. Vocal pitch regulation also involves the integration of auditory feedback in ongoing vocal motor control^4^,^9^,^10^, thus it is possible that the online control of vocal production can likewise benefit from perceptual learning. To date, this hypothesis has not been tested.

The aim of the present study was to examine whether auditory perceptual learning can lead to beneficial effects in the auditory-motor processing of perceived vocal pitch errors. Mandarin speakers were trained to perceive five Thai lexical tones while learning to associate pictures of objects with spoken words over 5 days. Before and after training, participants performed a vocal production task, where they produced sustained vowel sounds while hearing their voice unexpectedly shifted in pitch. We examined the behavioural and neural responses to pitch feedback perturbations during vocalization, and correlated them with learning performance. We expected that participants who received speech-sound training would exhibit significant changes in their vocal and cortical responses to pitch perturbations compared to participants who did not receive speech-sound training.

## Methods

### Subjects

Twenty-four students from Sun Yat-sen University in China participated in the present study. The participants were randomly assigned to the trained group (6 males and 6 females, 20–24 years of age) and the control group (5 males and 7 females, 18–23 years of age). These two groups did not significantly differ in age (*t* = 1.520, *p* = 0.143) or gender (χ^2^ = 0.168, *p* = 0.682). All subjects were native Mandarin speakers, right-handed, and had no history of speech, language, hearing, or neurological disorders. All subjects passed a pure-tone audiometric screening at the threshold of 25 dB hearing level (HL) that ranged from 500 to 4000 Hz for both ears. All subjects provided written consent in compliance with a protocol approved by the Institution Review Board of The First Affiliated Hospital at Sun Yat-sen University in China. The study was carried out in accordance with the approved guidelines.

### Sound-to-word learning program

**Figure 1:**
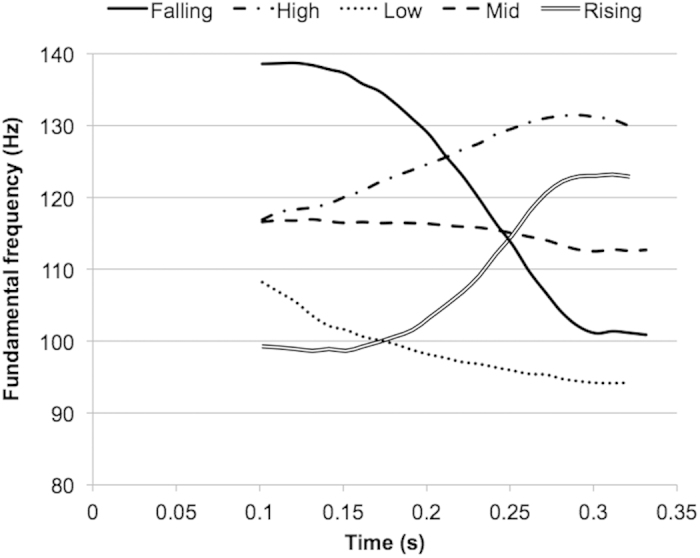
Pitch contours of the five lexical tones of Thai language (high, low, mid, falling, and rising) that were superimposed on three English pesudowords.

A sound-to-word learning paradigm, adopted from Wong and colleagues^28^,^29^,^30^, was used in the present study. Trained participants underwent a 5-day training during which they learned to associate speech stimuli with pictures of objects that were presented on a computer screen. To successfully learn the sound-picture pairings, participants had to be sensitive not only to the segmental features of the spoken words, but also to their suprasegmental features; namely, changes in pitch pattern within syllables, which mimicked the lexical tones of a tonal language. In a series of previous studies, English-speaking participants without previous experience with tonal languages were successfully trained to discriminate and identify Mandarin-Chinese-like tones using this sound-to-picture learning paradigm^28^,^29^,^30^. In the present study, instead of training participants who did not speak a tonal language to learn a tone system, we trained tonal language speakers whose native language was Mandarin Chinese to acquire an unfamiliar tone system that mimicked the Thai tone system. The Thai tone system differs from Mandarin in that Thai has five tones (low, mid, high, rising, and falling) (see Fig. 1), whereas Mandarin has four tones (high, rising, falling-then-rising, and falling).

The training stimuli consisted of three English pseudowords ([pæʃ], [nik], and [fæs]) with pitch patterns resembling five Thai tones, leading to a total of 15 words for participants to learn. The Pitch Synchronous Overlap and Add (PSOLA) method in Praat^31^ was used to generate the five pitch patterns for each of the three pseudowords. Each of the 15 pseudowords was then paired with a unique cartoon picture of an object (e.g. a dog, a potato). The 15 words were divided into three blocks, with one block for each base syllable presented with the five pitch patterns.

Participants in the trained group participated in two sessions per day over the course of 5 days with no more than a 2-day gap between each session. Each training session lasted about 30–45 minutes. Participants learned to associate the picture of an object presented on a computer screen with one of the 15 words, over the course of 10 sessions. Each session consisted of a training phase and a testing phase. In the training phase, the words in each block differed only in terms of lexical pitch. For each block, sound-picture pairing trials were presented 30 times with an inter-trial interval of 3s. For each trial, the cartoon picture of an object was shown on the screen and at the same time the corresponding speech token was played via the headphones. At the end of each block, participants were tested on the words they had just learned: each sound was played and then participants had to select the correct picture from five choices. If the participant selected a wrong picture, the correct picture would be displayed briefly before the presentation of the next test item.

After the training phase, the testing phase began during which participants heard the 15 trained words in a pseudorandom order and were asked to identify each word by selecting the correct picture from among 15 alternatives. The participants were given as much time as they needed to identify the words and no feedback was given during this testing phase. Memory for the word-picture associations was assessed by calculating a word identification score (WIS), the percentage of correct answers out of the 15 trained words presented during the testing phase.

### Vocal task

Before and after the 5-day sound-to-word learning program, participants in the trained group participated in an AAF-based vocal production task. Participants in the control group did not undergo the training, but they likewise performed the AAF-based vocal production task at time intervals equivalent to the participants in the trained group. During this task, participants sustained a phonation of the vowel /u/ for about 5–6 seconds at their habitual pitch and loudness level. Auditory feedback regarding their voice was randomly pitch-shifted downwards 5 times per vocalization. The duration of each pitch shift was 200 ms, and the first pitch shift was presented 500–1000 ms after vocal onset. The succeeding four pitch shifts occurred with an inter-stimulus interval (ISI) of 700–900 ms. Participants experienced three blocks of 20 vocalizations, with 100 pitch shifts per block, resulting in a total of 300 pitch-shift trials for each participant. During each block, the magnitude of the pitch shift was held constant at −40, −80, or −120 cents (−100 cents is equivalent to a downward shift of one semitone). The order of the three blocks was counterbalanced across participants.

### Data acquisition

Both trained and control participants performed the AAF-based vocal production task in a sound-attenuating booth. In order to partially mask the air-born and bone-conducted feedback, acoustic calibration was performed to ensure that voice feedback was heard with a sound pressure level (SPL) gain of 10 dB, relative to the participants’ vocal output. A dynamic microphone (model SM-306, Genuine Shupu) was used to record the voice signals, which were then amplified by a MOTU Ultralite Mk3 firewire audio interface. The vocal signals were then sent to an Eventide Eclipse Harmonizer, which pitch-shifted the amplified vocalizations based on MIDI commands sent by a custom-developed software program (Max/MSP, v.5.0 by Cycling 74) running on an iMac computer. Participants heard their pitch-shifted voice feedback through insert earphones (ER1-14A, Etymotic Research Inc.). Transistor-transistor logic (TTL) control pulses, generated by the Max/MSP software program, were used to signal the onset and offset of the pitch shifts. The delay between the time that the Harmonizer presented the pitch shifts to the participant, and the time that TTL control pulse was generated, was about 15 ms. The voice, feedback, and TTL control pulses were digitized at a sampling frequency of 10 kHz by a PowerLab A/D converter (model ML880, AD Instruments), recorded using LabChart software (v.7.0 by AD Instruments), and saved onto another iMac computer.

In addition to the acoustic signals, electroencephalograph (EEG) signals referenced to the vertex (Cz) were simultaneously recorded using a Geodesic Sensor Net with 64 scalp electrodes (Electrical Geodesics Inc., Eugene, OR). EEG signals were amplified with a Net Amps 300 amplifier (Electrical Geodesics Inc.) and digitized at a sampling frequency of 1 kHz. Individual sensors were adjusted to ensure that impedances were maintained below 50 kΩ throughout the recording^32^. EEG data from all channels were recorded using NetStation software (v.4.5, Electrical Geodesics Inc.) and saved onto a Mac Pro computer.

### Vocal data analysis

Vocal responses were measured using the event-related averaging techniques^33^. For each participant, voice fundamental frequency (F_0_) contours in Hertz were extracted from the voice signals using Praat^31^ and then converted to a cent wave in IGOR PRO (v.6.0, Wavemetrics Inc.) using the formula: cents = 100 × (12 × log_2_(F_0_/reference)), where the reference denotes an arbitrary reference note of 195.997 Hz (G4). All trials were segmented into epochs ranging from 200 ms before to 700 ms after the onset of the pitch shift. A visual inspection was performed to exclude any trials containing vocal interruptions or signal processing errors from further analyses. Finally, the artifact-free trials for each condition were averaged to generate an overall response for each participant. The latency of vocal response was determined as the time when the response exceeded 2 standard deviations (SDs) above or below the pre-stimulus mean following the onset of the pitch shift, and the magnitude was measured in cents by subtracting the pre-stimulus mean from the peak value of the voice contour following the response onset^33^.

### ERP data analysis

NetStation software was used for the off-line analyses of the EEG signals. EEG signals were band-passed filtered with a cut-off frequency of 1 to 20 Hz, and segmented into epochs with a window of 200 ms before and 500 ms after the onset of the pitch shift. Segmented trials were then inspected for artifact-contaminated trials caused by excessive muscular activity, eye blinks, and/or eye movements, using an Artifact Detection Toolbox in NetStation. Additional visual inspection was performed to ensure that the artifacts were adequately identified and rejected. Finally, artifact-free segmented trials were averaged, re-referenced to the average of electrodes on each mastoid, and baseline corrected for each condition. The amplitude and latency of N1 and P2 components measured at electrodes FC1, FCz, FC2, C1, Cz, C2, P1, Pz, and P2 were extracted for further data analyses. Pitch-shifting voice auditory feedback has previously been shown to affect the N1-P2 complex, and the cortical responses to pitch shifts are largest at those electrodes^34^,^35^.

### Statistical analyses

The magnitude and latency of the vocal responses and the N1-P2 complex were subjected to repeated-measures analysis of variance (RM-ANOVAs) in SPSS (v.16.0). Three-way RM-ANOVAs were used for the magnitude and latency of the vocal responses, in which session (pre- vs. post-training) and pitch-shift magnitude (−40, −80, and −120 cents) were chosen as within-subject factors, and group (trained vs. control) was chosen as a between-subject factor. The amplitude and latency of the N1-P2 complex were subjected to four-way RM-ANOVAs, including within-subject factors of session, pitch-shift magnitude, and site (FC1, FCz, FC2, C1, Cz, C2, P1, Pz, P2) as well as a between-subject factor of group. Subsidiary RM-ANOVAs were conducted if higher-order interactions reached significance. Probability values were adjusted using the Greenhouse-Geisser correction when the assumption of sphericity was violated, and corrected *p*-values were reported along with original degrees of freedom. In addition, Spearman correlation coefficients were used to examine the correlations between variables including vocal compensation (magnitude and latency), cortical responses (N1 and P2 amplitudes), and the sound-to-word learning performance (WIS).

## Results

### Word identification scores

**Figure 2:**
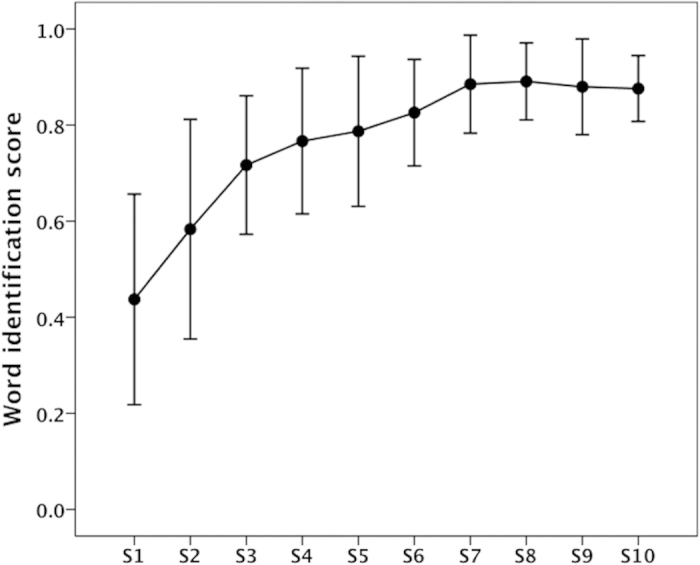
The learning curve indexed by the grand-averaged word identification scores (WISs) obtained at the end of each session across all participants (error bars represent standard deviation).

A WIS was calculated for each of the 10 training sessions. The averaged learning curve of the trained group across ten training sessions is shown in Fig. 2. At the end of the first training day (i.e., sessions 1–2), the averaged WIS was 51.02% (range: 17.80 ~ 90.00%). At the end of the last training day, subjects’ average WIS had improved to 87.78% (range: 66.70 ~ 96.70%). There was a significant increase in the WIS across the five training days (F(4, 44) = 46.426, p < 0.001). Bonferroni-adjusted comparison tests revealed significant improvement in learning between the first and all other training days (p < 0.001), between the second and the fourth (p = 0.001) and the fifth training days (p = 0.003), and between the third and the fourth training days (p = 0.042).

### Vocal responses

A three-way RM-ANOVA was performed on the latency of vocal responses to pitch-shifts, and the results revealed no significant main effect of session (F(1, 22) = 0.013, p = 0.911), pitch-shift magnitude (F(2, 44) = 0.408, p = 0.668), or group (F(1, 22) = 0.147, p = 0.705). No interactive effects were found to be significant either (p > 0.05).

**Figure 3:**
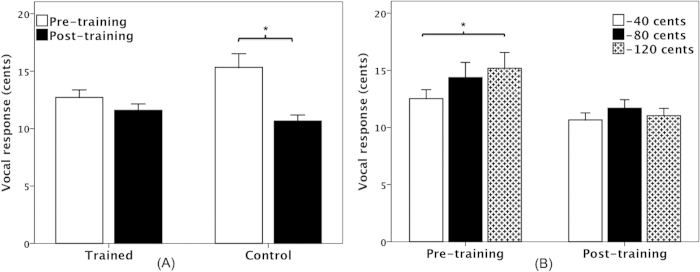
T-bar plots of the amplitude of vocal compensation (mean+standard error) at the pre-training and the post-training sessions as a function of group. (**A**) and pitch-shift magnitude (**B**). (A): The control group produced significantly smaller vocal responses at the post-training session as compared to the pre-training session (p = 0.007), while the trained group did not (p = 0.120). The trained group did not differ from the control group at the pre-training (p=0.232) or post-training session (p = 0.422). (**B**): There was a significant main effect of pitch-shift magnitude at the pre-training session (p = 0.015), where −120 cents pitch shifts elicited larger vocal responses than −40 cents pitch shifts. By contrast, main effect of pitch-shift magnitude did not reach significance at the post-training session (p = 0.163).

For response magnitudes, significant main effects of session (F(1, 22) = 13.841, p = 0.001) and pitch-shift magnitude (F(2, 44) = 3.360, p = 0.044) were found, but there was no main effect of group (F(1, 22) = 0.322, p = 0.576). A significant interaction was found between session and group (F(1, 22) = 5.186, p = 0.033). Follow-up RM-ANOVAs showed that the trained group did not differ from the control group at the pre-training (F(1, 22) = 1.511, p = 0.232) or post-training session (F(1, 22) = 0.671, p = 0.422). In addition, the control group produced significantly smaller response magnitudes at the post-training session relative to the pre-training session (F(1, 11) = 11.014, p = 0.007), but the main effect of session did not reach significance for the trained group (F(1, 11) = 2.838, p = 0.120) (see Fig. 3A).

There was also a significant interaction between session and pitch-shift magnitude (F(2, 44) = 3.922, p = 0.027). Follow-up RM-ANOVAs revealed a significant main effect of pitch-shift magnitude at the pre-training session (F(2, 44) = 4.595, p = 0.015), where −120 cents pitch shifts elicited larger responses than −40 cents pitch shifts (p = 0.026). At the post-training session, however, the main effect of pitch-shift magnitude did not reach significance (F(1, 22) = 1.894, p = 0.163) (see Fig. 3B).

### ERP results

**Figure 4:**
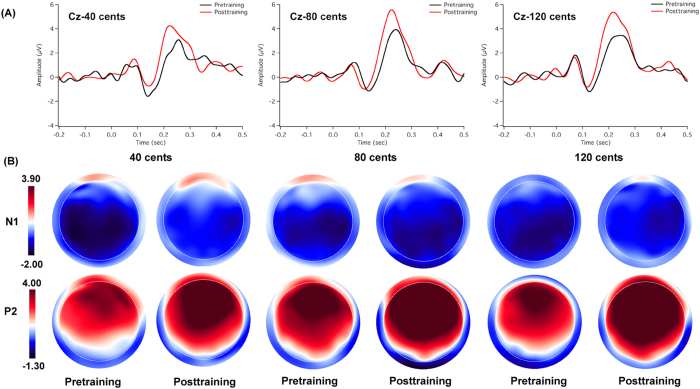
Grand-averaged ERP waveforms. (**A**) and topographical distributions of N1 and P2 components (**B**) in response to −40, −80, and −120 cents pitch shifts for the trained group. The black and red solid lines denote the cortical responses at the pre-training and post-training session, respectively.

**Figure 5:**
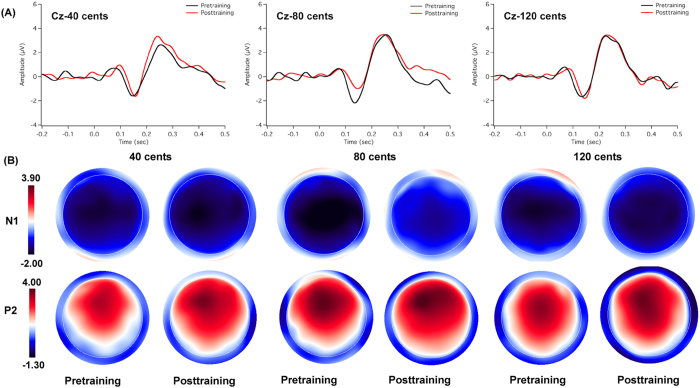
Grand-averaged ERP waveforms. (**A**) and topographical distributions of N1 and P2 components (**B**) in response to −40, −80, and −120 cents pitch shifts for the control group. The black and red solid lines denote the cortical responses at the pre-training and post-training session, respectively.

Figs. 4, 5 show the grand-averaged ERP waveforms (top) and topographic distributions (bottom) of N1 and P2 amplitudes in response to pitch-shifts as a function of pitch-shift magnitude and session for the trained (Fig. 4) and control groups (Fig. 5). As can be seen in Fig. 4, the trained group exhibited increased P2 responses, but decreased N1 responses (less negative) following training. Pitch shifts of −80 and −120 cents pitch shifts elicited larger P2 amplitudes than −40 cents pitch shifts. N1 latencies, particularly in the case of −80 and −120 cents pitch shifts, became shorter at the post-training session relative to the pre-training session. In contrast, the cortical responses at the pre- and post-training sessions were similar for the control group (see Fig. 5). This training-induced difference can also be seen in the topographical distributions of N1 and P2 amplitudes. In addition, cortical responses in the frontal and central areas appeared to be larger than those in the parietal areas as shown in Fig. 4B and Fig. 5B.

**Figure 6:**
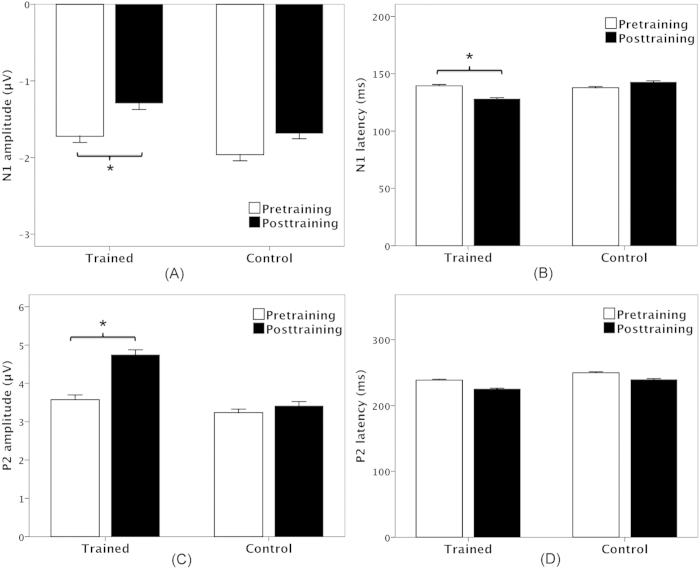
T-bar plots of the amplitude (left) and latency (right) (mean+standard error) of N1 (top) and P2 (bottom) components as a function of session and group. The white and black T-bars denote the responses at the pre-training and post-training session, respectively. The trained participants produced significantly smaller N1 amplitudes (p = 0.028), shorter N1 latencies (p = 0.027), and larger P2 amplitudes (p = 0.021) at the pre-training session as compared to the post-training session.

A four-way RM-ANOVA performed on the N1 amplitudes revealed significant main effects of session (F(1, 22) = 5.269, p = 0.032) and site (F(8, 176) = 5.534, p < 0.001), whereas the main effects of pitch-shift magnitude (F(2, 44) = 0.141, p = 0.835) and group (F(1, 22) = 0.539, p = 0.471) did not reach significance. A significant interaction was found between site and group (F(8, 176) = 4.154, p = 0.024). A follow-up RM-ANOVA conducted on the trained group data revealed significantly decreased N1 amplitudes at the post-training session relative to the pre-training session (F(1, 11) = 6.405, p = 0.028) (see Fig. 6A). There was no main effect of site (F(8, 88) = 2.663, p = 0.078) or pitch-shift magnitude (F(2, 22) = 0.386, p = 0.685). For the control group, N1 amplitude did not differ as a function of session (F(1, 11) = 1.162, p = 0.304) or pitch-shift magnitude (F(2, 22) = 0.018, p = 0.982), although there was a significant main effect of site (F(8, 88) = 6.301, p = 0.013), which was primarily driven by N1 amplitudes that were smaller at the parietal electrodes than at the frontal and central electrodes.

For N1 latency, main effects of pitch-shift magnitude (F(2, 44) = 5.269, p < 0.001) and site (F(8, 176) = 3.696, p = 0.037) reached significance, whereas main effects of session (F(1, 22) = 1.394, p = 0.250) and group (F(1, 22) = 1.946, p = 0.177) did not. Pitch shifts of −40 cents elicited significantly longer N1 latencies than did both the −80 (p = 0.003) and −120 cents pitch shifts (p < 0.001) stimuli. A significant interaction was found between session and group (F(2, 44) = 8.798, p = 0.007). A follow-up RM-ANOVA conducted on the trained group data revealed significantly decreased N1 latencies (F(1, 11) = 6.480, p=0.027) at the post-training session relative to the pre-training session (see Fig. 6B). In contrast, N1 latencies did not differ as a function of session (F(1, 11) = 2.368, p = 0.152) for the control group.

A four-way RM-ANOVA performed on the P2 amplitudes revealed significant main effects of session (F(1, 22) = 8.133, p = 0.009), pitch-shift magnitude (F(2, 44) = 3.479, p = 0.040), and site (F(8, 176) = 86.733, p < 0.001), whereas the main effect of group did not reach significance (F(1, 22) = 2.493, p = 0.100). A significant interaction was observed between session and group (F(1, 22) = 4.448, p = 0.047). A following-up RM-ANOVA conducted on the trained group data revealed significantly increased P2 amplitudes at the post-training session relative to the pre-training session (F(1, 11) = 7.304, p = 0.021), whereas there was no systematic change of P2 amplitudes as a function of session (F(1, 11) = 0.875, p = 0.370) for the control group (see Fig. 6C). In addition, the trained group did not differ from the control group at the pre-training session (F(1, 22) = 0.422, p = 0.523), whereas greater P2 amplitudes were produced by the trained group when compared to the control group at the post-training session (F(1, 22) = 5.615, p = 0.027).

The P2 latency results revealed a significant main effect of session (F(1, 22) = 9.729, p = 0.005), as P2 latencies became significantly shorter at the post-training session relative to the pre-training session (see Fig. 6D). The main effect of pitch-shift magnitude also reached significance (F(2, 44) = 5.035, p = 0.011); −120 cents pitch shifts elicited shorter P2 latencies than −40 cents pitch shifts (p = 0.009). However, there was no significant main effect of site (F(8, 176) = 0.690, p = 0.700) or group (F(1, 22) = 4.235, p = 0.052).

In order to examine whether changes in vocal/cortical responses to pitch shifts were related to the sound-to-word learning, Spearman correlation coefficients were used to calculate the correlation between WISs and the amplitude of the N1-P2 components. The results revealed a significant correlation between the post-pre difference for the WISs and the post-pre difference for the mean P2 amplitude across the three pitch-shift magnitudes (ρ = 0.658, p = 0.020), indicating a positive relationship between enhanced cortical processing of pitch errors in voice auditory feedback and improved auditory perceptual skills. However, this relationship was not observed for N1 amplitudes (ρ = −0.053, p = 0.871). Similarly, there was no significant correlation between the WISs and the magnitude or latency of the vocal responses. In addition, no significant correlations existed between the vocal and cortical responses to pitch shifts.

## Discussion

The present study examined whether perceptual learning of new speech sounds alters the auditory-motor processing of perceived vocal pitch errors in auditory feedback. The results revealed that Mandarin speaking participants who learned to identify the meaning of pseudowords that differed in their segmental and suprasegmental aspects produced significantly larger P2 amplitudes, smaller N1 amplitudes, and shorter N1 latencies when they heard pitch errors in their voice auditory feedback. In particular, there was a significant positive correlation between WISs and P2 amplitudes, which suggests a cause-effect relationship between speech-sound learning and auditory cortical processing of pitch feedback errors. At the behavioural level, vocal compensation magnitudes were consistent across the pre- and post-training sessions for the trained group, while there was a significant decrease in the magnitude of vocal compensation for the control group. These findings provide evidence that auditory-motor processing of pitch feedback errors can be shaped by speech-sound learning at the cortical level. Furthermore, these results suggest that functional changes to auditory processing that results from the perceptual learning of new speech sounds may generalize to the processing of auditory feedback, which in turn facilitates the detection of mismatches between intended vocal pitch productions and auditory feedback.

Our results are complementary to two recent behavioural studies pairing speech perceptual training with speech motor learning, despite methodological differences across the studies^19^,^27^. Both Lametti *et al.*^19^ and Shiller *et al.*^20^ showed significant changes in the speech motor adaptation to feedback perturbations during speech production as a consequence of training in the perception of the vowels contrasts (e.g. /ε/-**/**æ/). The present findings are also in line with other studies that assessed the effects of perceptual training on limb motor control^21^,^22^,^23^,^24^. For example, brief periods of somatosensory discrimination learning have been shown to improve the rate and extent of sensorimotor learning^22^, and result in changes in the functional connectivity of frontal motor regions which have been linked to perceptual learning^23^. The present work extends these findings by linking perceptual learning in speech to cortical changes in sensorimotor integration during voice control. These findings support the hypothesis that auditory perceptual training can drive changes in speech motor control.

The trained participants exhibited a significant training-induced increase in P2 response to pitch feedback errors. Moreover, P2 amplitudes were predictive of speech-sound learning performance, which suggests a relationship between changes in the cortical processing of pitch errors during speech and the proficiency of speech-sound learning. This finding is consistent with previous studies that have reported enhanced P2 responses following training on the acoustic discrimination of speech syllables or pure tones^36^,^37^,^38^. Also, there was a significant decrease in N1 amplitudes following speech-sound learning, which is consistent with the results of a magnetoencephalography (MEG) study that reported a decrease in N1m amplitudes after participants were trained to discriminate small differences in the frequency and intensity of tones^39^. It is noteworthy that the N1-P2 complex recorded in the AAF paradigm is believed to reflect both the detection and correction of feedback errors during vocal pitch regulation. For example, relative to passive listening to the playback of one’s own voice, active vocalization elicits suppressed N1 responses to pitch-shifted voice auditory feedback introduced at utterance onset^40^,^41^, while pitch shifts that occur at mid-utterance result in an enhancement of P2 responses^34^,^42^. Moreover, there is evidence showing that N1/P2 amplitudes and/or latencies are significantly correlated with the magnitude of vocal compensation for pitch shifts^43^,^44^. In light of these findings, changes in the N1-P2 complex observed in the present study most likely reflect training-related neural effects on the auditory-motor processing of feedback errors in vocal pitch regulation.

Interestingly, the trained participants did not exhibit systematic changes in their vocal responses as a function of the training sessions. This finding is partly in line with one study of melody discrimination training that improved perceptual skills, but did not lead to enhanced vocal accuracy^45^; this suggests that there is a dissociation between auditory perception and vocal production. According to the sensorimotor integration model^46^, good auditory discrimination skills are not associated with good vocal production, because auditory representations of sounds are incorrectly mapped on to motor representations. As such, vocal production cannot benefit from corrective feedback because motor corrections are in turn mapped on to inappropriate vocal targets. If this hypothesis were to be true, however, the vocal compensations should not have differed as a function of session for both the trained and the control participants. Nonetheless, our results revealed a significant decrease in the vocal response produced by the control participants. An alternative interpretation, therefore, is that speech-sound learning may result in improved vocal stability. There is evidence that people with more accurate pitch discrimination (such as singers, relative to non-singers) rely more on internal models (i.e. the mapping between motor commands and the resultant sensory consequences) to regulate their vocal production and generate more stable vocal output^9^,^47^. In light of this account, enhanced discrimination of linguistic pitch contours induced by speech-sound learning may have resulted in an increased reliance on internal models for the trained participants, which led to the improved stability in vocal motor control.

Regarding the significant decrease in the size of the vocal responses produced by the control participants, we cannot provide specific explanations because no studies have evaluated the test-retest reliability of vocal response to pitch shifts. We speculate that, relative to participants who underwent the speech-sound learning, the control participants may weight more heavily on their auditory feedback to ensure the detection of feedback errors. In this case, an adaptation effect resulting from repeated exposure to the same pitch shifts may have occurred, which led to the decreased vocal compensation. We cannot exclude the possibility, however, that the change in the magnitude of vocal responses was due to the sample size or the test-retest time interval for the control participants, and further studies should be conducted to test these factors.

Having established that cortical responses to pitch feedback errors are modulated by speech-sound learning, we may ask how perceptual learning of speech can produce transfer effects that are generalized to the neural processing of vocal pitch monitoring. Training-induced enhancement of P2 responses is often considered to result from an increase in the number of neurons that respond to an auditory stimulus, or an increase in the synchrony of the depolarization of stimulus sensitive neurons^38^, which increases the efficiency of the neural networks involved in pitch processing. It has been suggested that the neurons that underlie the P2 component receive input from cortical areas in the Sylvian fissure^48^. The posterior Sylvian fissure is thought to be a part of a network in the auditory system that performs coordinate transformation between auditory and motor representations^49^. Therefore, enhanced P2 responses observed in the present study may index the training-induced enhancement of the coordinated neural activity that reflects the interaction between the auditory and motor systems in voice control.

Although the perceptual training led to larger P2 amplitudes in response to vocal pitch feedback errors, smaller amplitudes and shorter latencies were observed for the N1. The N1 component has several neural generators in the primary and secondary auditory cortices^48^ and is thought to index pre-attentive detection of a mismatch between incoming auditory stimuli and the memory trace of the previous sensory input into the auditory system^50^. Similar to the reduction of N1 responses observed in this study, decreased brain activity in auditory regions has been reported in several training-related fMRI studies. For example, Jancke *et al.*^51^ observed significantly decreased brain activity in the planum temporale, planum polare, and sulcus temporalis superior after pitch discrimination training. Zatorre *et al.*^52^ also observed decreased brain activity in the right posterior auditory cortex when participants learned to discriminate micro-melodies. Decreased neural activity may be attributed to the reallocation of neural resources in information encoding^52^. Specifically, fewer neuronal units are required to encode the same level of information following training, leading to more efficient encoding of auditory information. In line with this interpretation, training participants to discriminate linguistic-related pitch stimuli may lead to increased efficiency in the neural encoding of pitch information by reducing the neuronal resources that are recruited for the detection of pitch errors in voice auditory feedback, which may be indexed by decreased amplitudes and shorter latencies of N1 amplitude that we observed in the present study.

Training-induced plasticity has been well documented in subjects actively performing a task associated with the training stimuli^19^,^27^,^52^,^53^,^54^. In the present study, however, we were able to elicit neurophysiological changes that reflect changes to the integration of auditory feedback during ongoing vocal pitch control by training participants to discriminate linguistic-related pitch contours. We favor the interpretation of a transfer effect, which refers to the phenomenon that active training associated with a particular task can influence performance on another unrelated task. In our study, the perceptual learning of new speech sounds resulted in the facilitation of the cortical processing of feedback errors during vocal pitch regulation. Transfer effects have been demonstrated in a number of other studies. For example, musical training can influence not only the processing of music, but also other cognitive processes in domains such as language^55^,^56^. There is also evidence that working memory training leads to the improvement of fluid intelligence, which is the ability to solve problems independently of previously acquired knowledge^57^.

It has been argued that transfer effects resulting from learning are mainly due to overlapping networks or shared cognitive mechanisms. Considerable neural convergence has been found between the speech production and perception systems. For example, the STG is an important area for the detection of feedback errors during vocal production^4^,^9^,^10^, while the motor and premotor cortices are active while listening to speech or musical rhythms^13^,^58^. According to the dual auditory processing model^16^, the speech perception and production systems are interdependent, in which feedforward projections from motor preparatory networks in the inferior frontal cortex and premotor cortex interface with feedback signals from auditory cortex via the inferior parietal lobule. These findings indicate an overlapping of neural networks involved in speech production and perception, which allows the two systems to operate together and influence each other. In light of this hypothesis, these overlapping processes and associated neural substrates may make contribute substantially to the successful transfer of training gains to the cortical processing of vocal pitch regulation.

One primary limitation of the present study was the use of a no-contact control group. This approach allowed us to rule out any test-retest improvements, but it is potentially vulnerable to the confounding effects of expectancy. That is, trained participants may have made more of an effort to improve their performance to produce a measurable improvement on post-training performance^59^. A stronger design would involve the comparison of the current training paradigm to a relatively easy pitch discrimination task as well as a pitch task involving unrelated stimuli in order to determine whether gains made by the trained participants were solely a result of training and not a result of more practice with the experimental environment or an awareness of experimenter expectation.

Overall, our findings provide evidence that the auditory-motor processing of feedback errors during vocal pitch regulation can be shaped by learning new speech-sound associations. The transfer effects of the speech perceptual learning, reflected as an enhancement of P2 and suppression of N1, as well as more stable vocal compensation in response to pitch-shifted voice auditory feedback, suggests that perceptual training may facilitate the neural mechanisms underlying the online control of vocal production. In conjunction with previous studies^18^,^19^,^60^, our results point to bidirectional influences of the auditory and motor systems on the perception and production of speech.

## Additional Information

**How to cite this article**: Chen, Z. *et al.* Transfer Effect of Speech-sound Learning on Auditory-motor Processing of Perceived Vocal Pitch Errors. *Sci. Rep.* **5**, 13134; doi: 10.1038/srep13134 (2015).
